# Predicting the Initial Treatment Response to Transarterial Chemoembolization in Intermediate-Stage Hepatocellular Carcinoma by the Integration of Radiomics and Deep Learning

**DOI:** 10.3389/fonc.2021.730282

**Published:** 2021-10-21

**Authors:** Jie Peng, Jinhua Huang, Guijia Huang, Jing Zhang

**Affiliations:** ^1^ Department of Oncology, The Second Affiliated Hospital, Guizhou Medical University, Kaili, China; ^2^ Department of Minimal Invasive Interventional Therapy, Sun Yat-Sen University Cancer Center, State Key Laboratory of Oncology in South China, Guangzhou, China; ^3^ Department of Medical Imaging Center, Nanfang Hospital, Southern Medical University, Guangzhou, China

**Keywords:** hepatocellular carcinoma, machine learning, deep learning, treatment response, TACE

## Abstract

**Objectives:**

We aimed to develop radiology-based models for the preoperative prediction of the initial treatment response to transarterial chemoembolization (TACE) in patients with hepatocellular carcinoma (HCC) since the integration of radiomics and deep learning (DL) has not been reported for TACE.

**Methods:**

Three hundred and ten intermediate-stage HCC patients who underwent TACE were recruited from three independent medical centers. Based on computed tomography (CT) images, recursive feature elimination (RFE) was used to select the most useful radiomics features. Five radiomics conventional machine learning (cML) models and a DL model were used for training and validation. Mutual correlations between each model were analyzed. The accuracies of integrating clinical variables, cML, and DL models were then evaluated.

**Results:**

Good predictive accuracies were showed across the two cohorts in the five cML models, especially the random forest algorithm (AUC = 0.967 and 0.964, respectively). DL showed high accuracies in the training and validation cohorts (AUC = 0.981 and 0.972, respectively). Significant mutual correlations were revealed between tumor size and the five cML models and DL model (each *P* < 0.001). The highest accuracies were achieved by integrating DL and the random forest algorithm in the training and validation cohorts (AUC = 0.995 and 0.994, respectively).

**Conclusion:**

The radiomics cML models and DL model showed notable accuracy for predicting the initial response to TACE treatment. Moreover, the integrated model could serve as a novel and accurate method for prediction in intermediate-stage HCC.

## Introduction

Hepatocellular carcinoma (HCC) is a major cause of cancer-related deaths worldwide (1). Although liver transplantation, surgical resection, and local ablation are radical curative operations in early-stage HCC, some patients in intermediate or advanced stages are ineligible for curative surgery (2–4). Apart from surgery, particularly for intermediate-stage patients, transarterial chemoembolization (TACE) is still the standard treatment modality following the National Comprehensive Cancer Network (NCCN) clinical practice guidelines (5–7).

Recent studies have reported that the initial treatment response is an indicator of a favorable clinical prognosis, such as better progression-free survival and overall survival (8–11). However, the development of a precise model for predicting the initial response to TACE therapy is desired, and radiomics is a promising method that involves the extraction of several quantitative features from radiology images, which could be feasibly used (12, 13). Previous studies have shown that conventional machine learning (cML) based on radiomics could be used to significantly predict clinical outcomes in cancers (14–18). In our previous studies, radiomics models could effectively predict microvascular invasion and progression-free survival before hepatectomy (19).

Deep learning (DL), including convolutional neural networks, as an emerging method for image classification, has been receiving increasing attention (20, 21). In HCC studies, these DL models can be trained and used for tumor detection, staging, and prognosis using radiology images (22–25). Similarly, a DL model based on computed tomography (CT) images could be used to predict prognosis to TACE (26–28). However, the integration of radiomics and DL models has not been reported for TACE, and its accuracy is unclear.

In our study, we extracted several radiomics features from radiology images and used five cML methods to predict the initial treatment response to TACE in HCC patients and created a DL model to train these CT images. Correlations between the five cML models, tumor size, and the DL model were further analyzed. By integrating tumor size, radiomics cML, and DL, precise ensemble models were developed and tested. This work has implications regarding the clinical use of artificial intelligence because it can be used repeatedly, non-invasively, and at a low cost.

## Materials and Methods

### Study Design and Patients

This was a retrospective study of 310 patients with Barcelona Clinic Liver Cancer stage B HCC who underwent conventional TACE between February 2015 and December 2020. The patients were recruited from Nanfang Hospital (n=139), Sun Yat-sen University Cancer Center (n=130), and the Second Affiliated Hospital of Guizhou Medical University (n=41). The inclusion criteria were radiologically or pathologically confirmed HCC, initial TACE treatment, and hepatic-arterial CT imaging availability within 7 days before treatment and 30 days after treatment. Patients who underwent locoregional or whole-body therapy were excluded. This study was approved by the Institutional Review Boards of the Second Affiliated Hospital of Guizhou Medical University, Nanfang Hospital and Sun Yat-sen University Cancer Center. All informed consents were signed and we conducted in accordance with the Declaration of Helsinki in the whole process.

### TACE Procedure and Response Evaluation

TACE was performed under local anesthesia using the traditional femoral approach. Our method is consistent with a previously reported practical standard method for Asian countries (29). TACE was performed under the guidance of digital subtraction angiography (Allura Xper FD 20, Philips) through the left and right hepatic arteries directly through the arteries supplying blood to the tumor when technically feasible. Hepatic arteriography, performed using a 5 Fr (RH or Yashiro) catheter, was first used to assess the location, number, size, and blood supply of the target tumor. The embolic emulsion agent, including epirubicin (30–60 mg), lobaplatin (30–50 mg), and lipiodol (10–30 mL), was injected into the artery supplying the tumor through a 2.7/2.8 Fr microcatheter. Thirty days after treatment, according to the modified Response Evaluation Criteria in Solid Tumors (ver. 1.1), the initial response to TACE was classified as complete response (CR), partial response (PR), stable disease (SD), or progressive disease (PD). Initial treatment response and non-response were strictly defined as CR + PR and SD + PD after the first course of TACE therapy.

### Manual Segmentation of the Regions of Interest

Contrast-enhanced CT (CECT) scans were performed as described in the **Supplementary Material**. The CECT images were downloaded using the Picture Archiving and Communication System (PACS). ITK-SNAP (version 3.6, https://sourceforge.net/projects/itksnapx64/) could only import medical image-related formats, such as DICOM and NITFI formats. We selected the tool from the main toolbar to annotate along the three axes. The tumor area was confirmed by hepatic-arterial, portal venous, and delayed-phase CT images. Two senior radiologists who were blinded to the treatment results manually segmented the three-dimensional (3D) regions of interest (ROIs) of HCC using ITK-SNAP. After completing the annotation of each layer, we could update the ROI and see the labeled 3D image in the bottom left window.

### Extracting Radiomics Features and Reproducibility Analysis

MATLAB 2014b (https://ww2.mathworks.cn/) was used to standardize and reconstruct the segmented 3D ROI image. The thickness of the layer was 1 mm. Python 3.6 (https://www.python.org/downloads/release/python-360/) was used to install the package and extract the radiomics features in 3D images (https://github.com/Radiomics/pyradiomics) (30). These values included the texture, shape, size, and wavelet transform of the CT images. The interclass correlation coefficients (ICCs) between the two observers were used to evaluate the repeatability of extracting radiomic features from 50 CT images in the training cohort. ICCs greater than 0.75 indicated favorable consistency in the radiomics extraction.

### Recursive Feature Elimination and Building the cML Models

**Figure 1:**
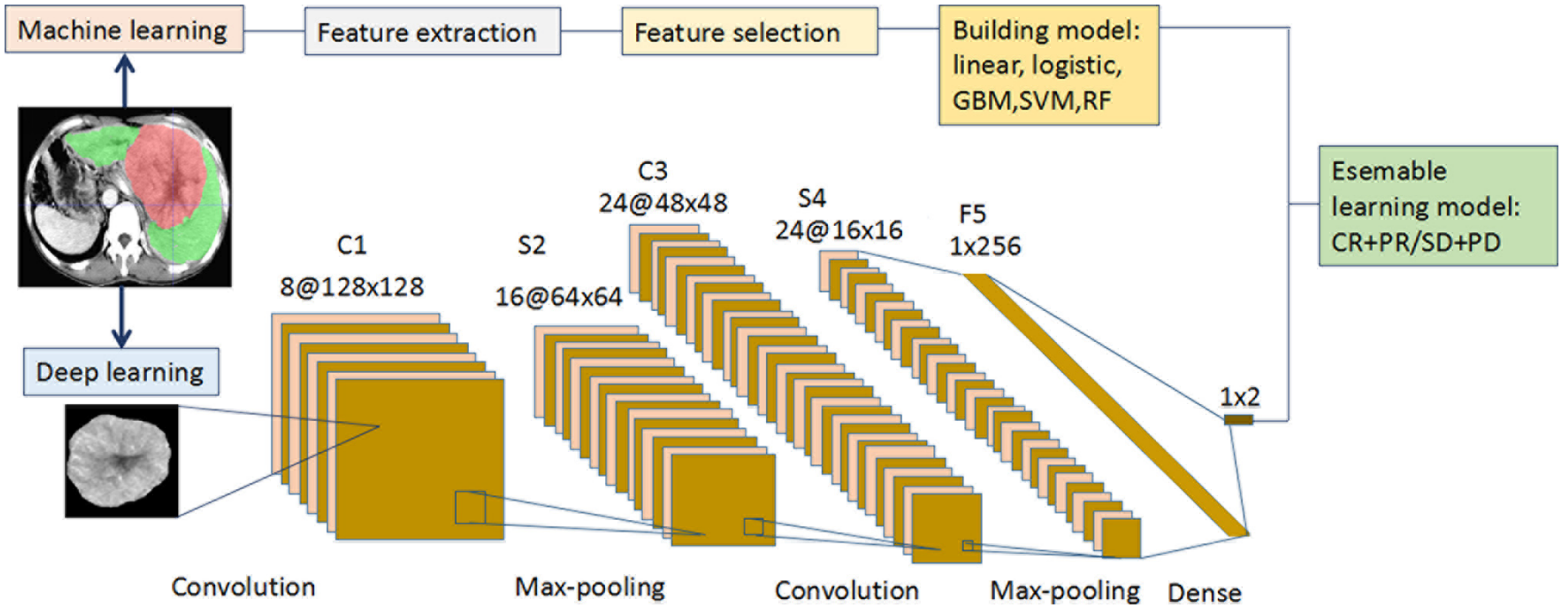
Flowchart for the integration of the machine learning and deep learning models. (i) The radiologists manually segmented the 3D ROIs of HCC using ITK-SNAP. Thereafter, 1167 features were extracted from the hepatic arterial CT images based on the “pyradiomics” package of python. Redundant radiomics features were then eliminated by ICCs. Using RFE and 5-fold cross-validation, the final features were selected and the five radiomics models were built using different machine learning algorithms. (ii) A CT image and mask of ROI manually segmented from the largest tumor layer was resized and output as 224 × 224 ×3 from each patient in the two cohorts. Augmentation techniques were used to enlarge the training CT image dataset, and “new” big data were generated. The deep learning framework included two convolutions, two max-poolings, and one dense layer. The final output layer was a softmax classifier. The optimizer was Adam, with a learning rate of 0.001 and batch size of 16. All the layers were standardized and the L2 regularization was set to 0.000001. The activation function of RELU was set as alpha = 0.1. (iii Each of the five machine learning models (linear, logistic, GBM, SVM, and RF) was integrated with the deep learning model to predict the initial treatment response to TACE. CT, computed tomography; GBM, gradient boosting machine; HCC, hepatocellular carcinoma; ICC, intraclass correlation coefficient; RELU, reconstructed linear units; RF, random forest; RFE, recursive feature elimination; ROI, region of interest; SVM, support vector machine; TACE, transarterial chemoembolization.

Recursive feature elimination (RFE) selects features by recursively reducing the size of the examined feature set (**Figure 1**). First, the prediction model is trained on the original features, and each feature is assigned a weight. Features that have the minimum absolute weight are then recursively removed from the feature set until the number of remaining features reaches the desired number of features. Random forest function and 5-fold cross-validation sampling were used in the RFE. RFE was performed with the “caret” R package. Five cML algorithms including linear, logistic, support vector machine (SVM), gradient boosting machine (GBM), and random forest (RF) were employed. Based on the 14 selected radiomics features, five radiomics models were then built by R packages (“C50,” “randomForest,” “caret,” and “e1071”).

### Data Preprocessing for Deep Learning

The intensity values of the CT images were mapped to [0, 1] for preparing the data for training deep learning. We defined the classified labels as CR + PR and SD + PD. A CT image and mask of the ROI manually segmented from the largest tumor layer in all patients was output. Based on an in-house algorithm, we extracted one largest patch with a size of 224 × 224 × 3 from every patient in the two cohorts. To avoid potential bias because for the unbalanced database, we used augmentation techniques to enlarge the CT image numbers, and new big data could be acquired for DL. The training patches were randomly distributed across the two classes by CT image augmentation, including level and vertical flip, vertical flip, level flip, and −90° and 90° rotation. Using this method, “new” training CT images were generated; this augmentation was not used in the other validation database.

### Deep Learning

Our DL framework is described as follows (**Figure 1**): the input layer was a CT image with a size of 224×224, and the C1 layer was a convolution layer. The S2 layer was a sampling layer, the feature map of each C1 layer was changed into a feature map with a size of 64×64, and the size of each sampling window was 2×2. The C3 layer was a convolution layer, and 24 feature maps with a size of 48×48 were obtained. Each feature map was the accumulation of the convolution of four feature maps in the S2 layer, the size of each convolution kernel was 5×5, and the convolution cores were 24×4. The S4 layer was a sampling layer. Each feature map of the C3 layer was sampled, 24 feature maps with a size of 16×16 were obtained, and the size of each sampling window was 22. The F5 layer transformed the 12 feature maps of the S4 layer into a vector containing 24×16×16 neurons, which were then converted into 12 feature maps when the back propagation calculation was performed. The output layer was a vector consisting of two neurons corresponding to the output of the two characters. The F5 and output layers formed a softmax classifier. The Adam optimizer was selected, with a learning rate of 0.001 and batch size of 16. All the layers were standardized, and the penalty factor of L2 regularization was set to 0.000001. The activation function of the reconstructed linear units was used, and its alpha was 0.1. The code was implemented as the GitHub dataset (https://github.com/Ashing00/Tensorflow_Lenet5). The training process was based on Tensorflow 2.0, and parameter tuning was performed in a Windows environment with a 2.6 GHz Intel Xeon Processor E5-2640V3 CPU, NVIDIA Pascal Titan X GPU, and 128 GB of RAM.

### Statistical Analysis

All statistical analyses were performed using R (version 3.5.0; R Core Team, 2018). χ²test was used to analyze the difference between clinical variables in training and validation cohorts. The performance of each radiomics model was evaluated in the training and validation cohorts using receiver operating characteristic (ROC) analysis. The optimal cutoff value for predicting treatment response was defined using the Youden index. The “pROC” package was used to plot the ROC curves. A 95% confidence interval (CI) for the area under the ROC curve (AUC) was calculated for all cohorts. The correlations between tumor size, each cML, and DL were analyzed by the “corrplot” package. Two-sided *P-*values < 0.05 were considered significant.

## Results

### Patient Characteristics and the Association With Treatment Response

In total, 139 and 171 patients were finally recruited in the training and validation cohorts, respectively (**Supplemental Figure S1**). The baseline patient characteristics are shown in **Supplemental Table S1**. A total of 121 (87.05%) and 152 (88.89%) patients in the training and validation cohort, respectively, were male. Further, 98 (70.50%) and 116 (67.83%) patients, respectively, were aged less than 60 years. Most patients (82.73% and 83.04%) had Child-Pugh A liver function. In the training *vs* validation cohorts, 66 (47.48%) *vs* 86 (50.29%) patients had high alpha-fetoprotein (AFP > 20 ng/mL) levels. Patients with large tumor size (> 5 cm) accounted for 83.45% and 89.47% of the population in the training and validation cohorts, respectively. Most patients had a low number of tumors (≤ 3) in the two groups (87.77% *vs* 80.70%). Eighty-three (59.71%) *vs* 104 (60.82%) patients in the two cohorts achieved CR + PR. There was no significant difference in treatment response rates between the two groups (each *P* > 0.05).

**Figure 2:**
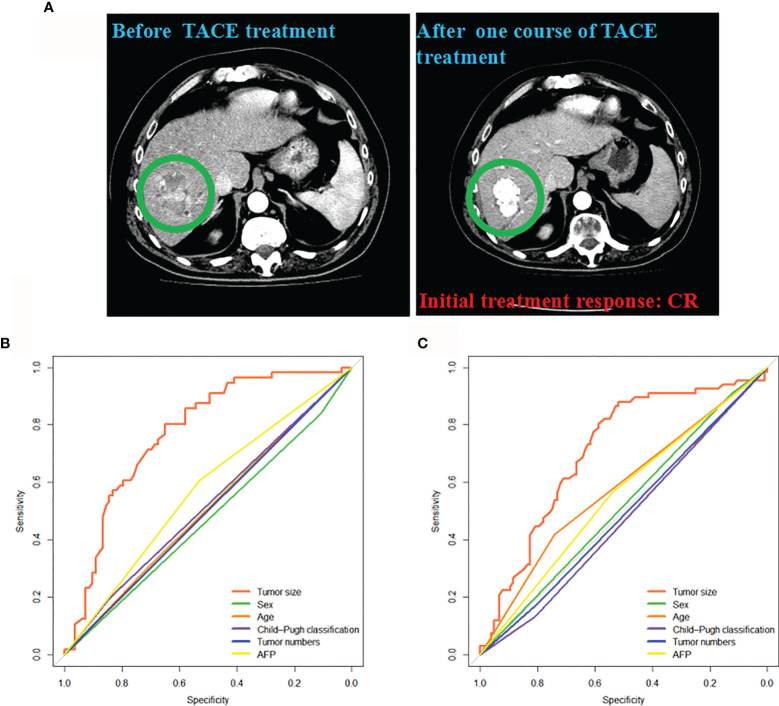
Associations between the clinical factors and initial treatment response to TACE. **(A)** The CT images from a patient acquiring CR after one course of TACE treatment were presented. Clinical factors predicting response (CR + PR) in the training **(B)** and validation cohorts **(C)**. CR, complete response; PR, partial response; TACE, transarterial chemoembolization.

The CT images showed the initial CR status after the first TACE treatment in a HCC patient (**Figure 2A**). The associations between clinical factors and treatment responses were further analyzed. Sex, age, Child-Pugh classification, AFP, and tumor numbers were not significant predictors of initial treatment response to TACE in intermediate-stage HCC in the training cohort (*P* = 0.601, 0.885, 0.691, 0.170 and 0.96, respectively) and validation cohort (*P* = 0.696, 0.667, 0.529, 0.552 and 0.511, respectively) (**Figures 2B, C**). The tumor size showed significant predictive value in the training and validation cohorts (AUC = 0.771, 95% CI: 0.693–0.850, *P* < 0.001 *vs* AUC = 0.709, 95% CI: 0.630–0.789, *P* < 0.001).

### Five Radiomics cML Models and the DL Model for Predicting Treatment Response

**Figure 3:**
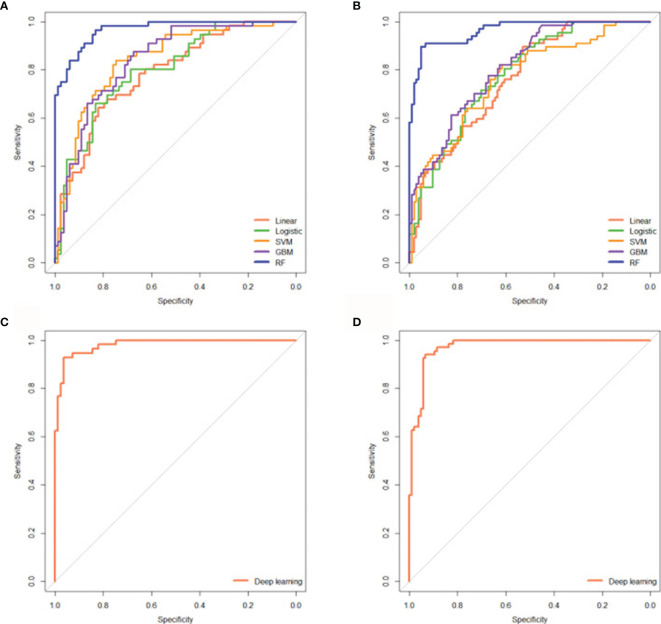
Five radiomics cML models and the DL model could precisely predict initial treatment response to TACE. ROC curves showing the predictive performance of the five cML models for estimating treatment response in the training **(A)** and validation cohorts **(B)**. ROC curves showing the predictive performance of the DL model for predicting treatment response in the training **(C)** and validation **(D)** cohorts. cML, conventional machine learning; DL, deep learning; ROC, receiver operating characteristic; TACE, transarterial chemoembolization.

A total of 1167 features were extracted from the hepatic-arterial 3D-CT images. A total of 457 pyradiomics features were eliminated in the ICC analysis. To acquire robust features, the remaining 710 features were subjected to feature selection using the RFE algorithm. Based on 5-fold cross-validation, 14 radiomics features were finally selected and used to build the five cML models (**Supplemental Table S2**). All machine learning models had significantly high prediction in the training and validation cohorts (each *P* < 0.001) (**Supplemental Table S3**). The simple linear model showed the lowest accuracy in the two cohorts (AUC = 0.784; 95% CI: 0.707–0.860, *P* < 0.001 *vs* AUC = 0.763, 95% CI: 0.693–0.833, *P* < 0.001). The logistic model was superior to the linear model in the training and validation groups (AUC = 0.801 *vs* 0.784 and 0.781 *vs* 0.763, respectively). For the three nonlinear models, RF showed better predictive accuracy than SVM and GBM in the two cohorts (AUC = 0.967 *vs* 0.841 and 0.839; and 0.964 *vs* 0.765 and 0.810, respectively) (**Figures 3A, B**). The three nonlinear models (SVM, GBM, and RF) had better predictive ability than the two linear models (linear and logistic). In the above engineered features analysis, the DL model demonstrated high accuracy in the training and validation cohorts (AUC = 0.981, 95% CI: 0.964–998, *P* < 0.001 *vs* AUC = 0.972, 95% CI: 0.951–0.993, *P* < 0.001) (**Figures 3C, D**). The five radiomics models and the DL model also showed high sensitivity and specificity in the two cohorts (**Supplemental Table S3**).

### Correlations Between Tumor Size, cML Models, and DL Model

**Figure 4:**
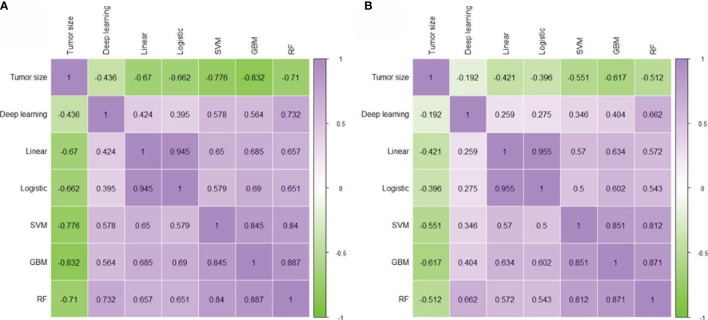
Correlation between tumor size, cML, and DL. Evaluating the mutual correlation between tumor size, the five radiomics cML models, and the DL model *via* correlation heatmaps in the training **(A)** and validation **(B)** cohorts. cML, conventional machine learning; DL, deep learning.

To evaluate the mutual correlation of tumor size, cML, and DL, we analyzed the predictive outcome of each model and found that tumor size, the cML models, and the DL model had significant correlations with one other (each *P* < 0.001) (**Figures 4A, B**). Tumor size was negatively associated with the other models in the two cohorts (each *P* < 0.001). We found that the linear model was most significantly correlated with the logistic model across the training and validation cohorts (r = 0.945, *P* < 0.001; r = 0.955, *P* < 0.001, respectively). In the nonlinear models, RF was most significantly correlated with GBM across the two cohorts (r = 0.887, *P* < 0.001; r = 0.871, *P* < 0.001, respectively). We also found that DL was most significantly associated with RF in the training and validation cohorts (r = 0.732, *P* < 0.001; r = 0.662, *P* < 0.001, respectively). There was a lower correlation between DL and the linear models than between DL and the nonlinear models in the training and validation cohorts (each *P* < 0.001) (**Figures 4A, B**).

### Evaluating Classifiable Accuracy Using the Integration Model

**Figure 5:**
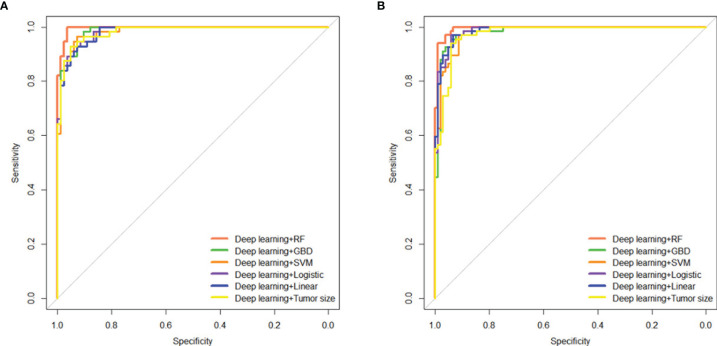
Integrating tumor size, cML models, and DL model to predict initial treatment response. Predictive performances of the ensemble model, including tumor size and the cML and DL models, are shown as ROC curves for the training **(A)** and validation **(B)** cohorts. cML, conventional machine learning; DL, deep learning; ROC, receiver operating characteristic.

To assess the predictive ability of combining DL with clinical factors or each model, we performed an integrative analysis of tumor size, cML, and DL. We found that DL combined with tumor size showed significantly high accuracy in the two cohorts (AUC = 0.983, 95% CI: 0.968–0.998, *P* < 0.001; AUC = 0.972, 95% CI: 0.951–0.993, *P* < 0.001) (**Supplemental Table S4**). The combination of DL and the simple linear model showed similar accuracy as the combination of DL and the logistic model in the training (AUC = 0.982 *vs* 0.984) and validation (AUC = 0.987 *vs* 0.986) cohorts. A combination of RF and DL showed the highest accuracy among the combinations of cML or tumor size with DL in the two cohorts (AUC = 0.995, 95% CI: 0.990–1.000, *P* < 0.001 *vs* AUC = 0.994, 95% CI: 0.987–1.000, *P* < 0.001) (**Figures 5A, B**).

## Discussion

In our study, the 14 most useful features based on RFE method were selected to develop the models. Additionally, the DL model was able to automatically extract many natural features from tumor ROI images. Using two distinct methods, we developed five radiomics cML models and a DL model to precisely predict the initial treatment response to TACE in the training and validation cohorts. Significant mutual correlations between the five cML models and the DL model were clearly elaborated in the two datasets. The combination of cML and DL can help to improve the prediction of treatment response compared to a single model, indicating that this method could act as a novel and useful tool for management of intermediate-stage HCC.

Regarding clinical factors, we found that tumor size, but not AFP, was significantly associated with the initial treatment response, which was consistent with a previous study (31). The high baseline levels of AFP (> 20 ng/mL) may not be related to the initial therapy response, but several studies have reported that the AFP decline was associated with treatment response and significantly improved median survival in intermediate-stage HCC after TACE therapy (32–34). Tumor number is an uncertain predictive biomarker of treatment response in different centers, and the outcome needs more studies for confirmation (35, 36). In addition to clinical variables, the predictive performance of the radiomics model in the study was similar to that of our linear and logistic models but was lower than that of the SVM, RF, and GBM models, which revealed that the optimal feature selection is important for building a precise model.

Unlike the engineered features model, the DL model as a novel method for image classification has been widely used in liver cancers (37–39). A DL model based on CT and gadoxetic acid-enhanced magnetic resonance imaging (EOB-MRI) seemed efficient for predicting microvascular invasion in HCC (40). A recent study established a DL model based on contrast-enhanced ultrasound (CEUS) to predict the initial treatment response of HCC patients to TACE using quantitative analysis (41). Our DL model from CT images has higher accuracies than CEUS models, indicating that CT images may be more useful than CEUS images in medical artificial intelligence. The predictive accuracy of our DL model was high, and this method could also be widely used in other HCC studies.

The potential relationships between radiomics cML and DL models were found significant mutual correlations between them. Moreover, the combination of cML and DL is an interesting topic and could most effectively use invisible radiology information. By integrating DL and radiomics analysis, pattern classification was able to achieve a high prediction in the benign and malignant pathology of gastrointestinal stromal tumors (42). Combining radiomics and DL signatures can be used to accurately predict lymph node metastasis in lung adenocarcinoma (43). However, the ensemble performances of five different cML models (such as SVM, GBM, and RF) in combination with DL have never been reported, and our study is the first to evaluate these performances in HCC patients receiving TACE therapy. The RF + DL model had the best predictive accuracy for the initial treatment response in HCC patients, indicating that optimal model integration could further improve the classification ability.

Our study has several limitations. First, our radiomics data were extracted from three medical centers, and the CT scanners in these centers were not the same and did not have similar parameters. Thus, the reproducibility of radiomics may have affected the robustness of the predictive model. Therefore, all CT images were standardized and reconstructed as 1-mm slices. Second, although the multicenter model analysis showed robust predictive performance, the study was conducted retrospectively, and the combined models need to be validated in a prospective study. Third, delineating the tumor ROI mostly depended on the interpreter’s experience of radiology, and manual methods required a lot of time. Future studies could develop an automatic segmentation model for liver tumors and minimize the discrepancies in radiomics features and DL training images. Finally, it is difficult to build a model with several lines of codes, particularly with regard to deep learning. Thus, it would be beneficial to identify an easy-to-use software that is free for clinical use.

In conclusion, the integration of five radiomics cML models and a DL model based on CT images is a noninvasive and low-cost but highly accurate model for predicting the initial treatment response to TACE in patients with intermediate-stage HCC. This integrating method could serve as a novel strategy to improve precise clinical decision-making in other malignant tumors.

## Data Availability Statement

The raw data supporting the conclusions of this article will be made available by the authors, without undue reservation.

## Ethics Statement

The studies involving human participants were reviewed and approved by the Second Affiliated Hospital,Guizhou Medical University. The patients/participants provided their written informed consent to participate in this study.

## Author Contributions

Conception and design: JP. Administrative support: JP. Provision of study materials and enrollment of patients: JH and JP. Collection and assembly of data: JP and JZ. Data analysis and interpretation: JP and JH. Manuscript writing: all authors. All authors contributed to the article and approved the submitted version.

## Funding

This work was supported by the Science and Technology Fund Project of Guizhou Provincial Health Commission [gzwjkj2019-1-077], Qian Dong Nan Science and Technology Program [qdnkhJz2019-026], Open Funds of State Key Laboratory of Oncology in South China [HN2020-02], Qian Dong Nan Science and Technology Program [qdnkhJz2020-013], Guizhou Medical University 2018 academic new talent cultivation and innovation exploration project [Grant No. 20185579-X], Science and Technology Foundation of Guizhou Province [Grant No. Qian ke he ji chu-ZK 2021, yi ban 454], National Nature Science Foundation of China (Grant Nos. 82060327), National Nature Science Foundation of China (Grant Nos. U20A20370) and Natural Science Foundation of Guangdong Province (Grant Nos. 2021A1515010370).

## Conflict of Interest

The authors declare that the research was conducted in the absence of any commercial or financial relationships that could be construed as a potential conflict of interest.

## Publisher’s Note

All claims expressed in this article are solely those of the authors and do not necessarily represent those of their affiliated organizations, or those of the publisher, the editors and the reviewers. Any product that may be evaluated in this article, or claim that may be made by its manufacturer, is not guaranteed or endorsed by the publisher.
