# Supplementary material for: Predicting the Initial Treatment Response to Transarterial Chemoembolization in Intermediate-Stage Hepatocellular Carcinoma by the Integration of Radiomics and Deep Learning
Source: Front Oncol. 2021 Oct 21;11:730282. doi: 10.3389/fonc.2021.730282 (PMC8566880; doi:10.3389/fonc.2021.730282)
Supplement: Supplementary file 1 [file DataSheet_1.docx]

**Supplementary Material**

**CT image acquisition**

Contrast-enhanced CT (CECT) images were acquired at three hospitals, using the following multi-detector row CT (MDCT). The Second Affiliated Hospital used SOMATOM (Siemens Medical Systems, Erlangen, Germany) scanner, Nanfang and Sun Yat-Sen University Cancer Center used Brilliance iCT 256 (Philips Healthcare, Cleveland, OH) scanner. Scanning parameters used in this study as following respectively: tube voltage, 120 kVp; detector collimation, 64×0.6 and 128×0.625 mm; field of view, 250-400 mm; pixel size, 512×512; rotation time, 0.5 s; slice interval, 0 mm; slice thickness, 5 mm; reconstructed section thicknesses, 1 mm.

CECT images were acquired after injection of 1.0 mL/kg contrast material (Ultravist 370, Bayer Schering Pharma, Berlin, Germany) into the antecubital vein at a rate of 2.0–3.0 mL/s using a power injector (Ulrich CT Plus 150, Ulrich Medical, Ulm, Germany); this was followed by a saline flush (20mL). Triphasic (hepatic arterial, portal venous, and delayed phase) CT images were acquired at 30, 60, and120 s after contrast material injection, respectively. The slice thickness of reconstructed arterial and portal venous phase images was 5 mm.

| **Table S1. Patient characteristics by study cohort.** | | | |
| --- | --- | --- | --- |
| Variable | Training cohort (n=139) | Validation cohort (n=171) | *P* value |
| Sex |  |  | 0.619 |
| Female | 18 (12.95%) | 19 (11.11%) |  |
| Male | 121 (87.05%) | 152 (88.89%) |  |
| Age (years) |  |  | 0.613 |
| ≤60 | 98 (70.50%) | 116 (67.83%) |  |
| >60 | 41 (29.50%) | 55 (32.17%) |  |
| Child–Pugh classification |  |  | 0.943 |
| A | 115 (82.73%) | 142 (83.04%) |  |
| B | 24 (17.27%) | 29 (16.96%) |  |
| AFP (ng/mL) |  |  | 0.622 |
| ≤20 | 73 (52.52%) | 85 (49.71%) |  |
| >20 | 66 (47.48%) | 86 (50.29%) |  |
| Tumor size (cm) |  |  | 0.119 |
| ≤5 | 23 (16.55%) | 18 (10.53%) |  |
| >5 | 116 (83.45%) | 153 (89.47%) |  |
| Tumor numbers |  |  | 0.092 |
| ≤3 | 122 (87.77%) | 138 (80.70%) |  |
| >3 | 17 (12.23%) | 33 (19.30%) |  |
| Treatment response |  |  | 0.843 |
| CR/PR | 83 (59.71%) | 104 (60.82%) |  |
| SD/PD | 56 (40.29%) | 67 (39.18%) |  |
| Note: *P* value is derived from the difference between the training and the validation cohorts. Abbreviations: AFP, alpha-fetoprotein; CR, complete response; PR, partial response; SD, stable disease; PD, progressive disease. | | | |

| **Table S2. Selecting radiomics features** |
| --- |
| **radiomics features (n = 14)** |
| wavelet.LLL_ngtdm_Busyness  wavelet.LLL_ngtdm_Complexity  squareroot_ngtdm_Strength  wavelet.LLL_glszm_LargeAreaLowGrayLevelEmphasis  wavelet.HLL_ngtdm_Strength  wavelet.LLL_glszm_GrayLevelVariance  original_ngtdm_Strength  wavelet.LLL_ngtdm_Contrast  wavelet.HLL_gldm_GrayLevelNonUniformity  wavelet.HLL_ngtdm_Coarseness  original_ngtdm_Complexity  logarithm_ngtdm_Busyness  wavelet.LLL_glrlm_GrayLevelNonUniformity  wavelet.HLL_ngtdm_Complexity |

| **Table S3. Performance of machine learning and deep learning in treatment response prediction.** | | | | |
| --- | --- | --- | --- | --- |
| **Groups** | **AUC (95%CI)** | **Sensitivity (95%CI)** | **Specificity (95%CI)** | ***P* value** |
| Training cohort |  |  |  |  |
| Linear | 0.784 (0.707-0.860) | 74.70 (63.96-83.61) | 69.64 (55.90-81.22) | < 0.001* |
| Logistic | 0.801 (0.727-0.874) | 72.29 (61.38-81.55) | 75.00 (61.63-85.61) | < 0.001* |
| SVM | 0.841 (0.775-0.908) | 74.70 (63.96-83.61) | 83.93 (71.67-92.38) | < 0.001* |
| GBM | 0.839 (0.774-0.905) | 71.08 (60.09-80.52) | 82.14 (69.60-91.09) | < 0.001* |
| RF | 0.967 (0.944-0.991) | 89.16 (80.41-94.92) | 89.29 (78.12-95.97) | < 0.001* |
| Deep learning | 0.981 (0.964-0.998) | 92.77 (84.93-97.30) | 94.64 (85.13-98.88) | < 0.001* |
| Validation cohort |  |  |  |  |
| Linear | 0.763 (0.693-0.833) | 65.38 (55.42-74.45) | 67.16 (54.60-78.15) | < 0.001* |
| Logistic | 0.781 (0.713-0.848) | 67.31 (57.41-76.19) | 73.13 (60.90-83.24) | < 0.001* |
| SVM | 0.765 (0.693-0.838) | 66.35 (56.42-75.32) | 71.64 (59.31-81.99) | < 0.001* |
| GBM | 0.810 (0.748-0.873) | 73.08 (63.49-81.31) | 70.15 (57.73-80.72) | < 0.001* |
| RF | 0.964 (0.939-0.988) | 93.27 (86.62-97.25) | 91.04 (81.52-96.64) | < 0.001* |
| Deep learning | 0.972 (0.951-0.993) | 94.03 (85.41-98.35) | 93.27 (86.62-97.25) | < 0.001* |
| Abbreviations: AUC, area under the curve; CI, confidence interval; SVM, support vector machine; GBM, gradient boosting machine; RF, random forest. **P* value < 0.05. | | | | |

| **Table S4. Performance of esemable learning in treatment response prediction.** | | | | |
| --- | --- | --- | --- | --- |
| **Groups** | **AUC (95%CI)** | **Sensitivity (95%CI)** | **Specificity (95%CI)** | ***P* value** |
| Training cohort |  |  |  |  |
| Deep learning+tumor size | 0.983 (0.968-0.998) | 95.18 (88.12-98.67) | 92.86 (82.71-98.02) | < 0.001* |
| Deep learning+Linear | 0.982 (0.966-0.997) | 93.98 (86.50-98.02) | 91.07 (80.38-97.04) | < 0.001* |
| Deep learning+Logistic | 0.984 (0.969-0.998) | 93.98 (86.50-98.02) | 91.07 (80.38-97.04) | < 0.001* |
| Deep learning+SVM | 0.983 (0.968-0.999) | 92.77 (84.93-97.30) | 96.43 (87.69-99.56) | < 0.001* |
| Deep learning+GBM | 0.986 (0.973-0.999) | 90.36 (81.89-95.75) | 98.21 (90.45-99.95) | < 0.001* |
| Deep learning+RF | 0.995 (0.990-1.000) | 96.39 (89.80-99.25) | 100.00 (93.62-100.00) | < 0.001* |
| Validation cohort |  |  |  |  |
| Deep learning+tumor size | 0.976 (0.958-0.994) | 90.38 (83.03-95.29) | 97.01 (89.63-99.64) | < 0.001* |
| Deep learning+Linear | 0.986 (0.975-0.998) | 93.27 (86.62-97.25) | 97.01 (89.63-99.64) | < 0.001* |
| Deep learning+Logistic | 0.987 (0.975-0.999) | 94.23 (87.87-97.85) | 97.01 (89.63-99.64) | < 0.001* |
| Deep learning+SVM | 0.980 (0.965-0.996) | 91.35 (84.21-95.97) | 97.01 (89.63-99.64) | < 0.001* |
| Deep learning+GBM | 0.982 (0.966-0.998) | 92.31 (85.40-96.62) | 97.01 (89.63-99.64) | < 0.001* |
| Deep learning+RF | 0.994 (0.987-1.000) | 93.27 (86.62-97.25) | 100.00 (94.64-100.00) | < 0.001* |
| Abbreviations: AUC, area under the curve; CI, confidence interval; SVM, support vector machine; GBM, gradient boosting machine; RF, random forest. **P* value < 0.05. | | | | |


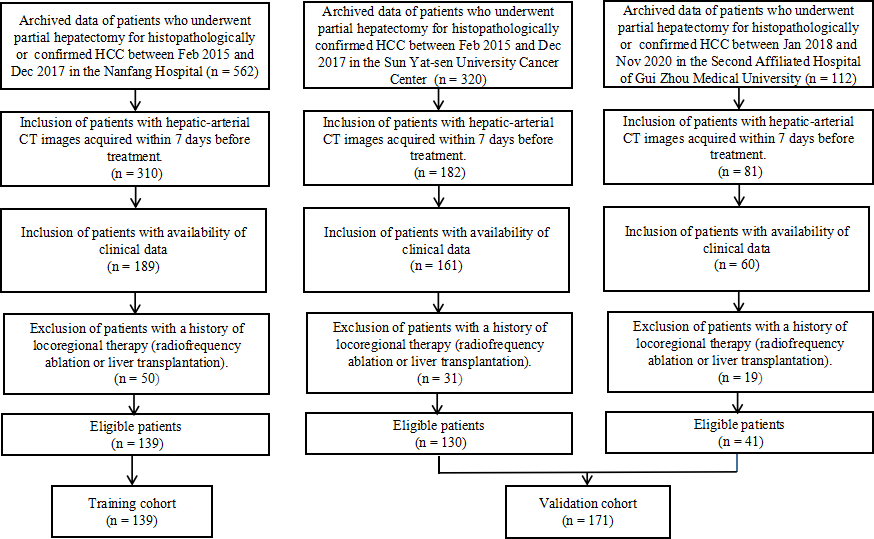


**Figure S1.** Flowchart shows the process of recruitment pathways for patients in training and validation cohorts.
